# Supplementary material for: Longitudinal Predictors of Self-Regulation at School Entry: Findings from the All Our Families Cohort
Source: Children (Basel). 2020 Oct 16;7(10):186. doi: 10.3390/children7100186 (PMC7602711; doi:10.3390/children7100186)
Supplement: Supplementary file 1 [file children-07-00186-s001.pdf]

Supplementary Material

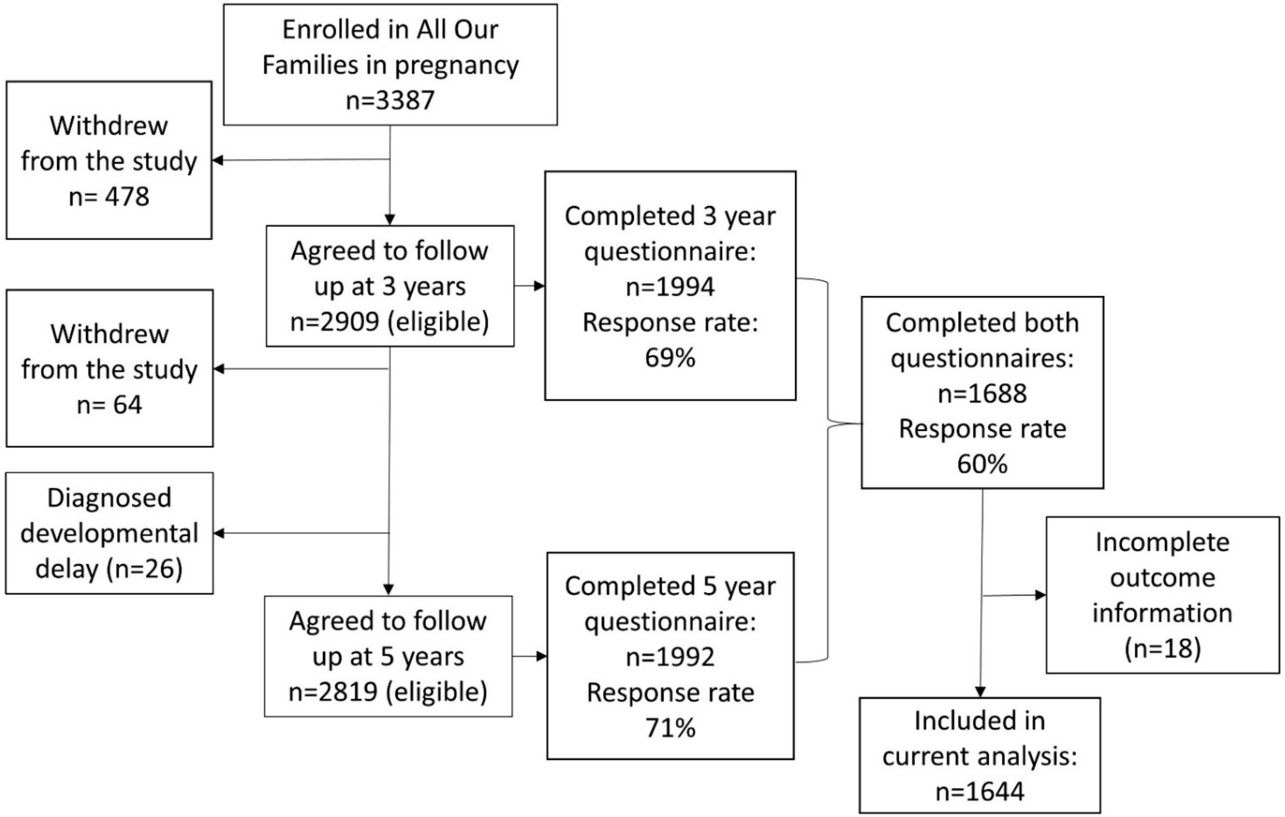

Figure S1. Flow chart for participant enrollment and response rate.

.Table S1: Adjusted Odd Ratios for poor self-regulation using continuous predictors.

**Models**	**Model 1: Overall poor self-regulation**		**Model 2: Severe poor self-regulation**		Model 3: Inattention		Model 4: Low Emotional Control		Model 5: Low Behavioral Control	
	**AOR**	**95% CI**	**AOR**	**95% CI**	AOR	95% CI	AOR	95% CI	AOR	95% CI
Increasing income	0.88	(0.82, 0.95)	0.97	(0.83, 1.14)	0.88	(0.80, 0.97)	0.93	(0.85, 1.02)	0.92	(0.83, 1.02)
Maternal anxiety symptoms @ 3yrs	**1.03**	**(1.01, 1.05)**	1.01	(0.98, 1.05)	**1.04**	**(1.02, 1.07)**	1.01	(0.98, 1.03)	**1.03**	**(1.00, 1.05)**
Maternal depressive symptoms @ 3yrs	1.01	(0.98, 1.03)	1.02	(0.98, 1.07)	0.99	(0.96, 1.02)	**1.03**	**(1.00, 1.06)**	1.01	(0.98, 1.04)
Childcare (10+ hours /wk)	1.29	(0.98, 1.71)	**1.79**	**(1.01, 3.16)**	**1.94**	**(1.36, 2.77)**	0.99	(0.70, 1.38)	1.29	(0.89, 1.86)
Male child	1.27	(0.98, 1.65)	**2.17**	**(1.22, 3.86)**	**1.86**	**(1.32, 2.62)**	1.05	(0.78, 1.43)	**1.58**	**(1.12, 2.23)**
Screen time (+ 1 hr)	**1.23**	**(1.03, 1.47)**	1.18	(0.82, 1.71)	**1.42**	**(1.13, 1.79)**	1.09	(0.89, 1.35)	1.12	(0.89, 1.41)
Higher hostile/ineffective parenting	**1.18**	**(1.13, 1.22)**	**1.19**	**(1.11, 1.28)**	**1.14**	**(1.09, 1.20)**	**1.16**	**(1.11, 1.21)**	**1.22**	**(1.16, 1.28)**
Higher positive parenting	1.02	(0.97, 1.08)	1.00	(0.89, 1.12)	1.03	(0.95, 1.10)	1.01	(0.95, 1.09)	1.02	(0.95, 1.10)

AOR: Adjusted Odds Ratio – adjusted for all variables in the table in addition to maternal age, maternal education, and child age. Original models included the following interaction terms: income*childcare, maternal anxiety*childcare, maternal depression*childcare, male child*hostile parenting, male child*positive parenting, screen time*hostile parenting, screen time*positive parenting). All interaction terms were dropped because they were not statistically significant (0.05) All statistically significant predictors (at 0.05) are bolded.
